# Supplementary figures and images for: Comparison between Two Adaptive Optics Methods for Imaging of Individual Retinal Pigmented Epithelial Cells
Source: Diagnostics (Basel). 2024 Apr 4;14(7):768. doi: 10.3390/diagnostics14070768 (PMC11012195; doi:10.3390/diagnostics14070768)

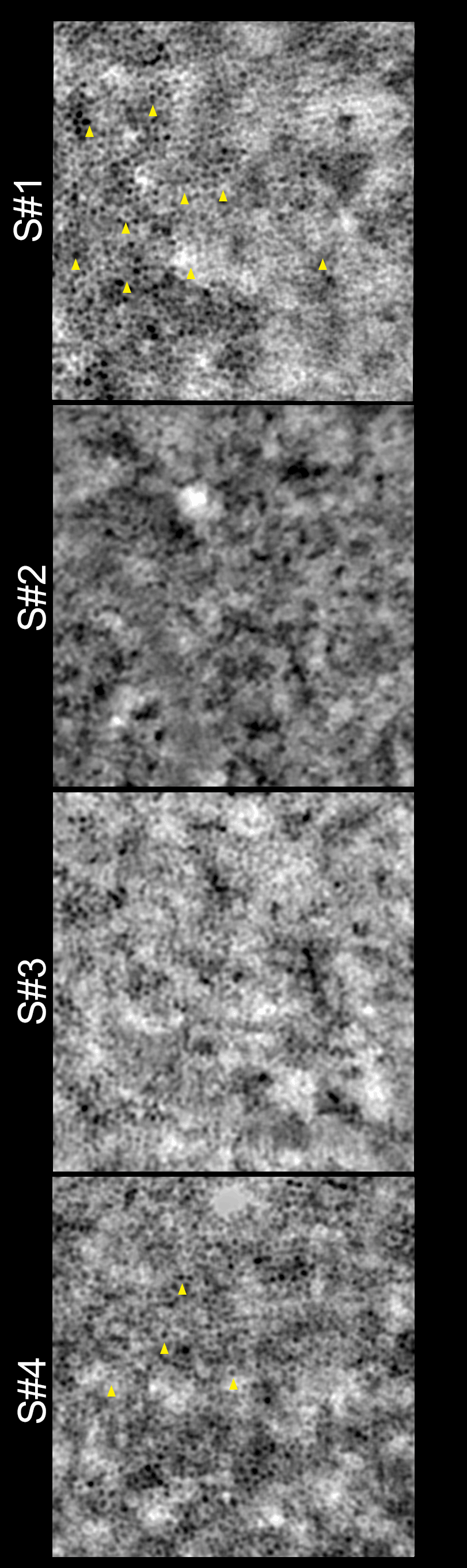

Supplement: Supplementary file 1 [file diagnostics-14-00768-s001.zip › diagnostics-2900697-supplementary.gif]
